# Plasma biomarker profiling of PIMS-TS, COVID-19 and SARS-CoV2 seropositive children – a cross-sectional observational study from southern India.

**DOI:** 10.1016/j.ebiom.2021.103317

**Published:** 2021-04-02

**Authors:** Aishwarya Venkataraman, Nathella Pavan Kumar, Luke Elizabeth Hanna, Sulochana Putlibai, M Karthick, Anuradha Rajamanikam, Kalaimaran Sadasivam, Balasubramanian Sundaram, Subash Babu

**Affiliations:** a ICMR-National Institute for Research in Tuberculosis, Chennai, India; b Kanchi Kamakoti CHILDS Trust Hospital, Chennai, India; c National Institutes of Health-National Institute for Research in Tuberculosis - International Center for Excellence in Research, Chennai, India; d LPD, NIAID, NIH, Bethesda, MD, United States

**Keywords:** COVID-19, PIMS-TS, MIS-C, SARS-CoV-2, Cytokines, Chemokines, Biomarker

## Abstract

**Background:**

SARS-CoV-2 infection in children can present with varied clinical phenotypes and understanding the pathogenesis is essential, to inform about the clinical trajectory and management.

**Methods:**

We performed a multiplex immune assay analysis and compared the plasma biomarkers of Paediatric inflammatory multisystem syndrome temporally associated with SARS-CoV-2 infection (PIMS-TS), acute COVID-19 infection (COVID-19), SARS-CoV-2 seropositive and control children admitted to a tertiary care children's hospital in Chennai, India. Pro-inflammatory cytokines, chemokines and growth factors were correlated with SARS-CoV-2 clinical phenotypes.

**Findings:**

PIMS-TS children had significantly elevated levels of cytokines, IFNγ, IL-2, TNFα, IL-1α, IFNα, IFNβ, IL-6, IL-15, IL-17A, GM-CSF, IL-10, IL-33 and IL-Ra; elevated chemokines, CCL2, CCL19, CCL20 and CXCL10 and elevated VEGF, Granzyme B and PDL-1 in comparison to COVID-19, seropositive and controls. COVID-19 children had elevated levels of IFNγ, IL-2, TNFα, IL-1α, IFNα, IFNβ, IL-6, IL-17A, IL-10, CCL2, CCL5, CCL11, CXCL10 and VEGF in comparison to seropositive and/or controls. Similarly, seropositive children had elevated levels of IFNγ, IL-2, IL-1α, IFNβ, IL-17A, IL-10, CCL5 and CXCL10 in comparison to control children. Plasma biomarkers in PIMS-TS and COVID-19 children showed a positive correlation with CRP and a negative correlation with the lymphocyte count and sodium levels.

**Interpretation:**

We describe a comprehensive plasma biomarker profile of children with different clinical spectrum of SARS-CoV-2 infection from a low- and middle-income country (LMIC) and observed that PIMS-TS is a distinct and unique immunopathogenic paediatric illness related to SARS-CoV-2 presenting with cytokine storm different from acute COVID-19 infection and other hyperinflammatory conditions.

**Research in context**

**Evidence before this study**

SARS-CoV-2 infection in children can present with varied clinical phenotypes. Children with PIMS-TS/MIS-C most commonly present with multi-organ dysfunction and elevated SARS-CoV-2 IgG antibodies, whereas children with acute COVID-19 infection present with a mild or asymptomatic illness. Understanding the pathogenesis is essential to help understand the differences and similarities between different clinical phenotypes and to inform about the clinical trajectory, so that optimal and effective treatment can be delivered.

**Added value of this Study**

To our knowledge this is the first study from a LMIC setting describing the plasma biomarker profile of children with different clinical spectrum of SARS-CoV-2 infection namely PIMS-TS, acute COVID-19 infection and asymptomatic seropositive. We were able to identify biosignatures that differentiates the clinical phenotypes distinctly and observed that the cytokine storm and hyperinflammation in PIMS-TS is different from that of acute COVID-19 and other hyperinflammatory conditions like Kawasaki Disease. Our results provide immunological basis for the use of current treatment modalities in PIMS-TS and emphasises the need for further research in identifying the optimal biological agent for treatment.

**Implications of all the available evidence**

The detailed analysis in our study provides additional information on the pathogenesis and may guide the clinicians in the assessment and management of children with PIMS-TS and acute COVID-19 infection in future.

Alt-text: Unlabelled box

## 1 Introduction

Severe acute respiratory syndrome coronavirus 2 (SARS-CoV-2) infection in children has generally been a mild or asymptomatic illness compared with adults [1]. However, Paediatric inflammatory multisystem syndrome temporally associated with SARS-CoV-2 infection (PIMS-TS) also known as Multisystem inflammatory syndrome in children (MIS-C), a potentially fatal disease, is now recognised as a distinct childhood illness related to SARS-CoV-2 [2,3]. Clinically, PIMS-TS occurs as a late manifestation of SARS-CoV-2 infection (3–6 weeks after) with many children presenting with multi-organ dysfunction and elevated SARS-CoV-2 IgG antibodies, whereas children with acute COVID-19 infection (COVID-19) present with a mild or asymptomatic illness. Given this diverse clinical presentation, there is an urgent need to understand the immunology and pathogenesis of SARS-CoV-2 infection in children, to help clinicians understand the differences and similarities between different clinical phenotypes (PIMS-TS, COVID-19, and asymptomatic seropositive), so that optimal and effective treatment can be delivered.

Studies on immunology of SARS-CoV-2 infections in children including PIMS-TS have mostly been from developed countries, [4–7] and there is currently no data from low- and middle-income countries (LMIC). We, therefore, performed a systemic level analysis of cytokines, chemokines, and growth factors in children with PIMS-TS and compared them to those in children with COVID-19, seropositive children and healthy controls presenting to a tertiary care children's hospital in Chennai, India, and aimed to define and identify important biomarkers involved in the immunopathogenesis of PIMS-TS and COVID-19.

## 2 Methods

### 2.1 Study design and participants

We performed an immunological analysis on plasma samples from children admitted to Kanchi Kamakoti CHILDS Trust Hospital (KKCTH), a tertiary children's hospital in Chennai, India from 1 June 2020 to 30 September 2020. Children of either sex, aged 12 months and under 18 years, needing hospitalisation for any condition, were included in this study, after caretaker's informed written consent. Blood was collected into EDTA tubes (BD Biosciences), plasma was obtained by centrifugation, and frozen at −80 °C until transferred to the National Institute for Research in Tuberculosis (NIRT), Chennai for serological and immunological analysis. Peripheral blood sampling in all children were done prior to receiving any immunomodulatory treatment. Multiplex assays on all samples were performed at the same time to limit batch effect. Study staff involved in immunological and serological assays were blinded to clinical data.

### 2.2 Clinical data

The KKCTH study team approached caregivers of all children admitted to hospital during the above mentioned period for participation in the study. Demographic, epidemiological, medical and laboratory data were collected on a standardised case report form. Anonymised data was shared with NIRT for statistical analysis. Acute COVID-19 disease and severity of COVID-19 was defined according to the Ministry of Health and Family Welfare (MOHFW) guidelines [8] issued by Government of India and children with PIMS-TS were diagnosed according to the Royal College of Paediatrics and Child Health (RCPCH) case definition for PIMS-TS [9].

### 2.3 SARS-CoV-2 RT-PCR test

SARS-CoV-2 real-time reverse-transcriptase polymerase chain reaction (RT-PCR) was performed by Indian Council of Medical Research (ICMR) approved laboratories. Results of RT-PCR were shared along with the clinical data by the KKCTH study team.

### 2.4 SARS-CoV-2antibodyassay

Serological and immunological assays were performed at National Institutes of Health -National Institute for Research in Tuberculosis - International center for Excellence in Research laboratory (NIH-NIRT-ICER), NIRT, Chennai. Antibodies were quantified in plasma using iFlash® SARS-CoV-2 IgG chemiluminescence antibody assay (CLIA) (YHLO Biotechnology Corporation, Shenzhen, China) according to the manufacturer's instructions. An antibody titre of ≥ 10 AU/ml was considered positive.

### 2.5 Multiplex assays

Circulating plasma levels of cytokines, chemokines and growth factors were measured by Luminex Magpix Multiplex Assay system (Bio-Rad, Hercules, CA) using Luminex Human Magnetic Assay kit 45 Plex (R & D systems). The lowest detection limits for cytokines were as follows: IFNγ, 5.7 pg/mL; IL-2, 3.6 pg/mL; TNFα, 12.4 pg/mL; IL-1α, 10.6 pg/mL; IL-1β, 3.5 pg/mL; IFNα, 3.9 pg/mL; IFNβ 3.25 pg/mL; IL-6, 9.0 pg/mL; IL-12, 18.5 pg/mL; IL-15, 2.5 pg/mL; IL-17A, 9 pg/mL; IL-3, 17 pg/mL; IL-7, 3.5 pg/mL; G-CSF, 8.4 pg/mL; GM-CSF, 18.4 pg/mL; IL-4, 1.1 pg/mL; IL-5, 6.2 pg/mL; IL-13, 31.8 pg/mL; IL-10, 32.2 pg/mL; IL-25, 18.4 pg/mL; IL-33, 13.8 pg/mL; IL-1Ra, 11.7 pg/mL. The lowest detection limits for chemokines were as follows: CCL2, 5.9 pg/mL; CCL3, 5.1 pg/mL; CCL4, 103.8 pg/mL; CCL5, 297 pg/mL; CCL11, 21.6 pg/mL; CCL19, 3.9 pg/mL; CCL20, 2.4 pg/mL; CXCL1, 19.1 pg/mL; CXCL2, 21.1 pg/mL; CXCL8, 1.4 pg/mL; CXCL10, 2.6 pg/mL and CX3CL1, 188 pg/mL. The lowest detection limits for growth factors were as follows: VEGF, 5.9 pg/mL; EGF, 8.6 pg/mL; FGF-2, 8.7 pg/mL; PDGF-AA, 5.2 pg/mL; PDGF-BB, 7.31 pg/mL; TGFa, 8.6 pg/mL; Flt-3 L, 22.9 pg/mL; Granzyme B (GZB), 4.9 pg/mL; PDL-1, 69.3 pg/mL; TRAIL, 22.5 pg/mL

### 2.6 Informed consent and ethical approval

Informed consent was obtained from caregivers, and assent was obtained from children where appropriate. The study was approved by the KKCTH CHILDS Trust Medical Research Foundation (CTMRF) ethics committee and the National Institute for Research in Tuberculosis Ethics committee. The study was also registered at Clinical Trials Registry India (CTRI/2021/01/030605).

### 2.7 Funding

This study was funded by National Institutes of Health -National Institute for Research in Tuberculosis - International center for Excellence in Research laboratory (NIH-NIRT-ICER), Chennai

### 2.8 Statistical analysis

For analysis, children were categorised into four groups as: COVID-19 (RT-PCR positive), PIMS-TS, Seropositive (IgG positive non PIMS-TS) and Control (both serology and RT-PCR negative). Continuous variables are presented as medians and interquartile ranges (IQRs), and categorical variables are reported as numbers and proportions. Comparison between the groups was performed using the Mann-Whitney *U* test. Geometric means (GM) were used for measurements of central tendency. Statistically significant differences between PIMS-TS, COVID-19, seropositive children and controls were analysed using the Kruskal-Wallis test with Dunn's multiple comparisons. Mann-Whitney test was used to compare cytokines concentrations between children with PIMS-TS needing Paediatric Intensive Care Unit (PICU) care and those who did not require PICU care as well as between COVID-19 children with mild disease and those with moderate to severe disease. *P* ≤ 0.05 was considered statistically significant and all tests were two sided. Analyses were performed using Graph-Pad PRISM Version 9.0 (GraphPad Software, CA, USA). Principal component analysis (PCA) and Spearman's correlation analysis were done using statistical software JMP 14.0 (SAS, Cary, NC, USA).

## 3 Results

### 3.1 Basic characteristics

We enrolled and performed the immunological assays on stored plasma samples of 145 hospitalised children (33 COVID-19, 44 PIMS-TS, 47 seropositive and 21 control children) from June to September 2020. The median age was 5 years (range 1 month - 17 years); and 58% (84/145) were male. All COVID-19 children were SARS-CoV-2 RT-PCT positive and all PIMS-TS children were seropositive (IgG). Of the 33 COVID-19 children, 22 (67%) presented with mild symptoms, 3 (9%) had moderate symptoms, 2 (6%) had severe symptoms needing PICU care and 6 (18%) children were asymptomatic. among the 47 seropositive non PIMS-TS children, 30 (64%) were admitted to the hospital for elective procedures; they therefore had no systemic symptoms. Further demographics and clinical presentation of COVID-19, PIMS-TS and seropositive children are described in Tables 1 and 2. Detailed clinical presentation of these children have been previously reported [10,11]. For comparison, we included 21 children with a similar median age (6 years, IQR: 1 – 15 years) and sex (male: 52%, 11/21), who were both SARS-CoV-2 RT-PCR negative and seronegative and presented to hospital for elective procedures (controls). They had no other co-morbid conditions and no history of COVID-19 contact.

**Table 1:** Characteristics of COVID-19, PIMS-TS and Seropositive (non PIMS-TS) children.

	COVID-19*n* = 33	PIMS-TS*n* = 44	Non PIMS-TS*n* = 47#	P value∞
Age median (years, IQR)	5 (1 – 17 yr)	7 y (1 – 14y)	4.4 y (1 – 17 y)	*p* < 0.05
Male n (%)	20 (60%)	19 (43%)	35 (74%)	*p* < 0.05
Serology IgG positive n (%)	0	44 (100%)	47 (100%)	
Underlying conditions n (%)	6 (19%)£	1 (2%)^a^	11 (23%)^c^	*p* < 0.05
Co-existing infections n (%)	4 (12%)£	5 (11%)^b^	6 (13%)^d^	*p* < 0.05
Median duration since proven or suspected COVID illness or contact (weeks, range)	NA	3 w (10 d – 4 w)	3.2 w (10 d – 5 w)ᵟ	*p* = 0.46
COVID-19 Symptoms n (%)				
* Fever*	28 (86%)	44 (100%)	17 (36%)	*p* < 0.05
* Gastrointestinal*	8 (23%)	37 (84%)	15 (32%)	*p* < 0.00001
* Respiratory*	10 (31%)	11 (25%)	16 (34%)	*p* = 0.17
* Mucocutaneous*	0	34 (77%)	0	*p* < 0.00001
* Asymptomatic*	6 (18%)	0	30* (64%)	*p* < 0.00001
Cardiovascular symptoms/signs				
* Hypotension*	0	23 (52%)	0	*p* < 0.00001
* Shock*	1	21 (48%)	2	*p* < 0.00001
* Coronary artery dilatation*	0	2	0	*p* < 0.00001
* Mycocardial dysfunction*	0	23 (52%)	0	
Laboratory parameters *n* = 29 *n* = 44 *n* = 31				
* CRP (< 5* *mg/L)*	5 (<5 – 181)	169 (39 – 473)	5 (<5 – 181) ¥	*p* < 0.00001
* Lymphocyte(/mm3)* *(1500 – 4000) median (IQR)*	2945 (650 – 12,000)	1386 (330 - 2200)	3890 (650 – 12,000)	*p* < 0.00001
* Neutrophils (/mm3)* *(1500 – 7000) median (IQR)*	3681 (120 – 13,160)	11,658 (9918 - 14,878)	6300 (120 – 13,160)	*p* < 0.00001
* Platelets (200 – 450)*x*10^9^/L median (IQR)*	278 (180 – 400)	110 (62 – 210)	327 (100 – 540)	*p* < 0.00001
* Sodium (135 –* 145 mmol*/l) median (IQR)*	136 (135 – 145)	134 (127 −138)	138 (135 – 148)	*p* < 0.00001
Median duration of stay	3 (1 – 7)	4.5 (3 – 12)	3 (1 – 10)	*p* = 0.0001
Treatment n (%)				
* IVIG*	0	37 (84%)	0	< 0.00001
* Steroids*	1 (4%)	32 (73%)	2 (4%)	< 0.00001
* PICU admission*	3 (10%)	23 (53%)	6 (11%) €	< 0.00001
* Antibiotics*	10 (31%)	39 (87%)	16 (31%)	< 0.00001
* Tocilizumab (*8 mg*/kg)*	0	2 (4%)	0	
Respiratory support n (%)				
* Mechanical Ventilation*	0	0	0	
* HHFNC*	1 (4%)	2 (3%)	2 (4%)	
* Oxygen*	3 (10%)	7 (16%)	5 (10%)	
Cardiovascular support n (%)				
* Inotropes*	0	23 (52%)	0	
* Fluid Bolus*	1 (4%)	28 (64%)	2 (4%)	

# Admitted for reasons other than COVID-19.

∞ p value between PIMS-TS, non PIMS-TS and COVID-19.

⁎ Admitted for elective surgery/procedures.

£ Details described in table 2.

¥ High CRP seen in children with co-infections.

ᵟ no history of COVID illness or contact in 4 children.

€ Admitted to PICU for reasons other than COVID-19. PICU: paediatric intensive care unit; HHFNC: high flow nasal cannula oxygen; IVIG: intravenous immunoglobulin, CRP: C - reactive protein.

a Atrial septal defect.

b Urinary tract infection, Scrub typhus, Enteric fever.

c Acute lymphoblastic leukaemia, Neurodevelopmental delay, Medulloblastoma, Inborn error of metabolism, Osteosarcoma, thalassemia, Haemolytic anaemia, Seizure disorder.

d Scrub typhus, Dengue viral fever, Tuberculosis, Brucellosis.

**Table 2:** Additional features of COVID-19 children (RT-PCR positive).

Total (n)	33
Male n (%)	20 (60%)
Age (Median, IQR)	5 (1 – 17 yr)
COVID-19 Clinical syndrome	n (%)
*Mild*	22 (67%)
*Moderate*	3 (9%)
*Severe*	2 (6%)
*Asymptomatic*	6 (18%)
Clinical Symptoms	
*Fever*	28 (86%)
*Respiratory*	10 (31%)
*Gastrointestinal*	8 (23%)
Underlying conditions (*n* = 6)	
*Acute lymphoblastic leukaemia*	2
*Neurodevelopmental delay*	1
*Nephrotic syndrome*	1
*Inborn error of metabolism*	1
*Iron deficiency Anaemia*	1
Co-existing infection/condition (*n* = 4)	
*Urinary tract infection*	2
*Brucellosis*	1
*Tuberculosis*	1

### 3.2 Plasma levels of cytokines are elevated in PIMS-TS and COVID-19 children

**Fig. 1:**
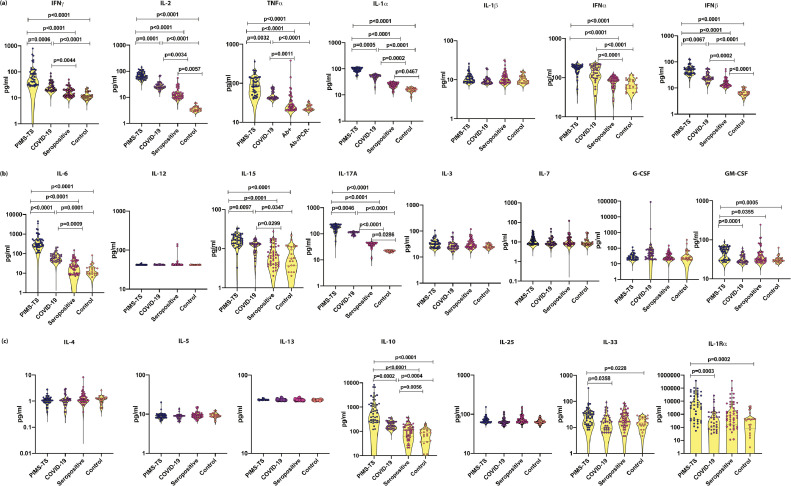
Elevated circulating levels of Type 1, Type 17 and other pro-inflammatory cytokines in children with PIMS-TS and COVID-19 (a) The plasma levels of IFNγ, IL-2, TNFα, IL-1α, IL-1β, IFNα, and IFN β, (b) IL-6, IL-12, IL-15, IL-17A, IL-3, IL-7, G-CSF and GM-CSF, (c) IL-4, IL-5, IL-13, IL-10, IL-25, IL-33 and IL-1Rα were measured in PIMS-TS (*n* = 44), COVID-19 (*n* = 33), seropositive (*n* = 47) and control children (*n* = 21). The data are represented as scatter plots with each circle representing a single individual. P values were calculated using the Kruskal-Wallis test with Dunn's post-hoc for multiple comparisons.

To ascertain if the cytokines can distinguish between different clinical phenotypes, we measured the plasma levels of Type 1, Type 2, Type 17, Type 1 IFNs and other pro inflammatory cytokines (Fig. 1). PIMS-TS children had significantly elevated levels of cytokines, IFNγ, IL-2, TNFα, IL-1α, IFNα, IFNβ, IL-6, IL-17A, GM-CSF, IL-10, IL-33 and IL-Ra in comparison to COVID-19, seropositive and control children. COVID-19 children had elevated levels of IFNγ, IL-2, TNFα, IL-1α, IFNα, IFNβ, IL-6, IL-17A and IL-10 in comparison to seropositive and/or control children. Similarly, seropositive children had elevated levels of IL-2, IL-1α, IFNβ and IL-17A in comparison to control children. We also observed that children with cardiac symptoms (*n* = 23) had predominantly elevated levels of TNFα, IL-6, IL-10, and IL-17A. Thus, plasma cytokines could clearly distinguish between PIMS-TS, COVID-19, seropositive and control children.

### 3.3 Plasma levels of CC and CXC chemokines are elevated in PIMS-TS and COVID-19 children

**Fig. 2:**
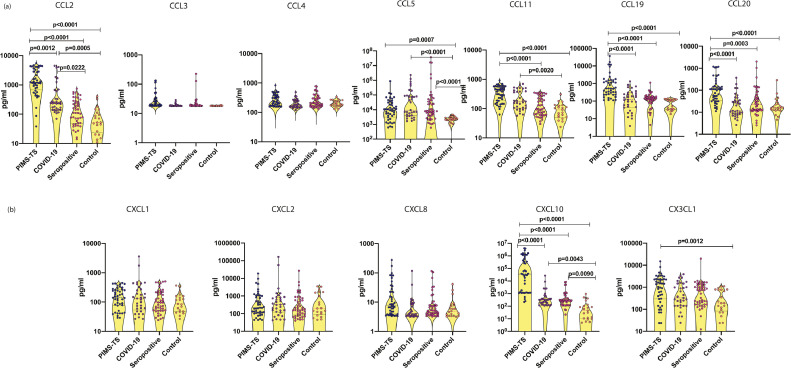
Elevated circulating levels of CC and CXC chemokines in children with PIMS-TS and COVID-19 (a) The plasma levels of CCL2, CCL3, CCL4, CCL5, CCL11, CCl19 and CCL20 (b) CXCL1, CXCL2, CXCL8, CXCL10 and CX3CL1 were measured in PIMS-TS (*n* = 44), COVID-19 (*n* = 33), seropositive (*n* = 47) and control children (*n* = 21). The data are represented as scatter plots with each circle representing a single individual. P values were calculated using the Kruskal-Wallis test with Dunn's post-hoc for multiple comparisons.

We measured the plasma levels of CC and CXC chemokines (Fig. 2) and observed that the levels of CCL2 CCL5, CCL11, CCL19, CCL20 and CXCL10 were significantly higher in PIMS-TS compared to COVID-19, seropositive and control children; COVID-19 children had elevated levels of CCL2, CCL5, CCL11 and CXCL10 in comparison to seropositive and/or control children; and seropositive children had elevated levels of CCL5 and CXCL10 in comparison to control children. PIMS-TS children with myocardial dysfunction and gastrointestinal manifestations, had markedly high levels of CCL-2, CCL-19 and CCL-20. Thus, plasma chemokines could distinguish between PIMS-TS, COVID-19, seropositive and control children.

### 3.4 Plasma levels of growth factors are elevated in PIMS-TS and COVID-19

**Fig. 3:**
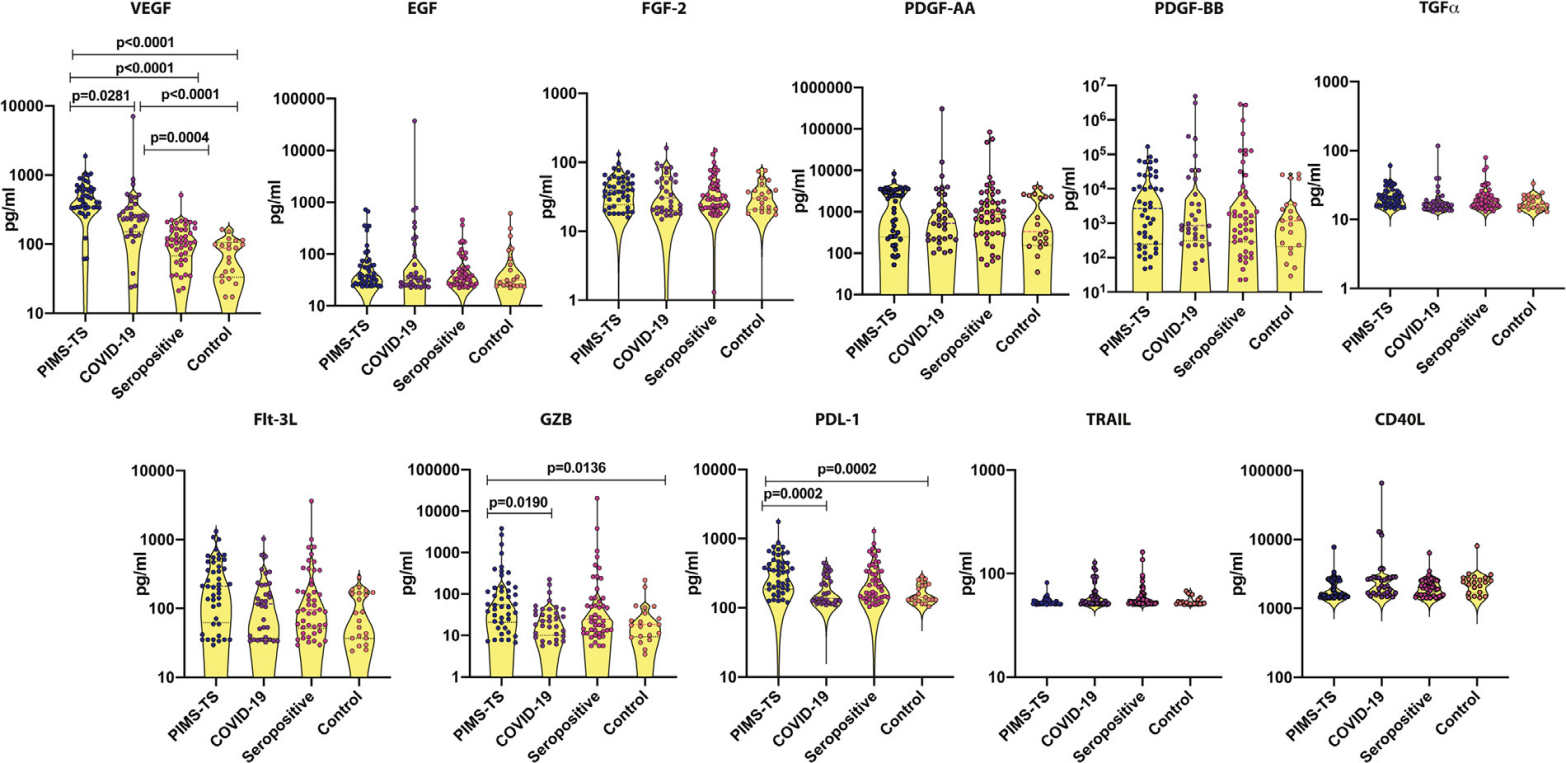
Elevated circulating levels of growth factors in children with PIMS-TS and COVID-19 The plasma levels of VEGF, EGF, FGF-2, PDGF-AA, PDGF-AB BB, TGFα, Flt-3 L, Granzyme B, PDL1, TRAIL and CD40L were measured in PIMS-TS (*n* = 44), COVID-19 (*n* = 33), seropositive (*n* = 47) and control children (*n* = 21). The data are represented as scatter plots with each circle representing a single individual. P values were calculated using the Kruskal-Wallis test with Dunn's post-hoc for multiple comparisons.

Analyses of growth factors (Fig. 3) showed significantly higher levels of VEGF, Granzyme B and PDL1 in PIMS-TS children compared to COVID-19, seropositive and control children. Similarly, COVID-19 children had elevated levels of VEGF in comparison to seropositive and control children. Thus, certain growth factors could distinguish between PIMS-TS, COVID-19, seropositive and control children. Elevated VEGF was found in all PIMS-TS children, signifying endothelial dysfunction.

### 3.5 Plasma inflammatory markers can robustly distinguish PIMS-TS from COVID-19 and seropositive and control children

**Fig. 4:**
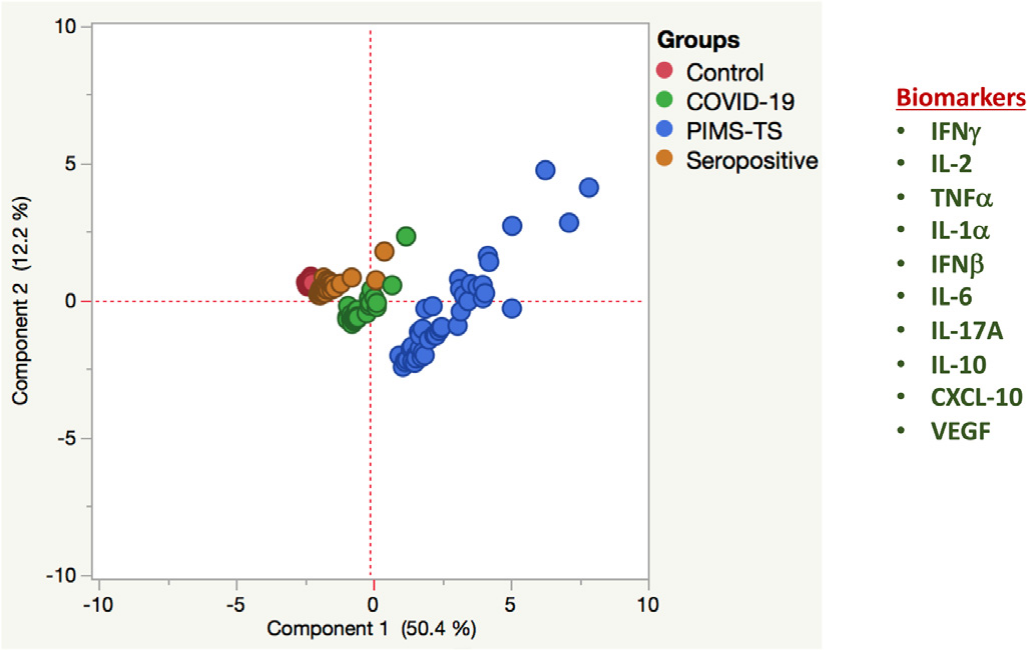
PCA analysis to estimate the discriminatory power of immune markers in children with PIMS-TS, COVID-19, seropositive and controls PCA (Principle component analysis) plot computing normalized ELISA data from plasma levels of selected cytokines, chemokines and growth factors in combination of four different experimental groups first PIMS-TS (Coloured in brown) vs COVID-19 (Coloured in red) vs seropositive (Coloured in blue) and controls (Coloured in green). The PCA shows the two principal components of variation, accounting for 12.2% (x -axis) and 50.4% (y -axis) (For interpretation of the references to color in this figure legend, the reader is referred to the web version of this article.).

We performed PCA (principal component analysis) of IFNγ, IL-2, TNFα, IL-1α, IFNβ, IL-6, IL-17A, IL-10, CXCL-10 and VEGF to determine the discriminatory power of plasma cytokines, chemokines and growth factors in distinguishing in children with PIMS-TS from COVID-19, seropositive and control children (Fig. 4). PCA evidently demonstrates the ability of these markers to differentiate PIMS-TS from COVID-19, seropositive and controls with elevated levels seen in PIMS-TS and COVID-19 children.

### 3.6 Plasma cytokines are markers of disease severity in PIMS-TS

**Fig. 5:**
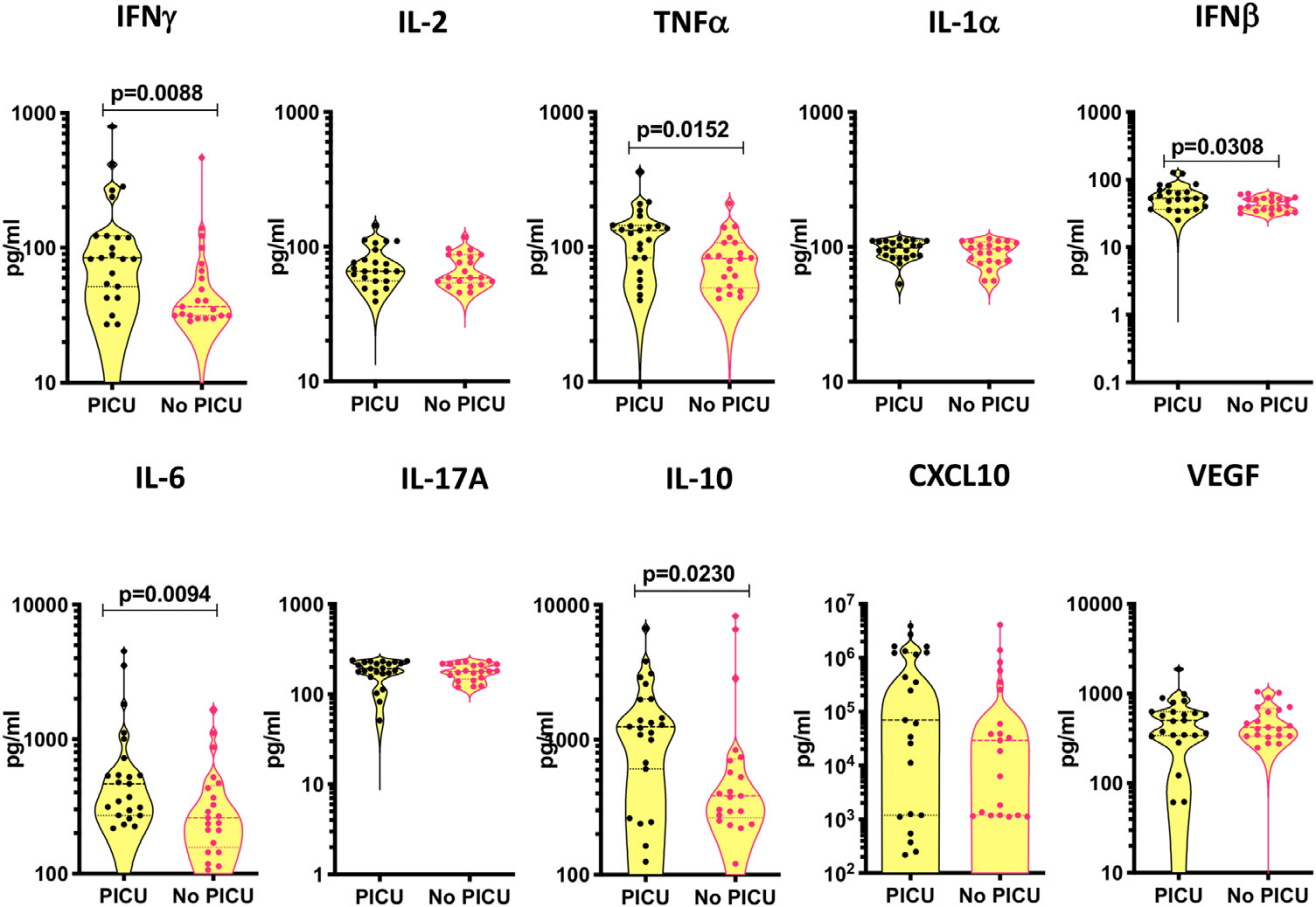
Circulating levels of cytokines, chemokines and growth factors in children with PIMS-TS requiring PICU care vs no PICU care. The plasma levels of IFNγ, IL-2, TNFα, IL-1α, IFNβ, IL-6, IL-17A, IL-10, CXCL10 and VEGF were measured in children with PIM-TS requiring PICU care (*n* = 23) and children in ward [who did not require PICU care] (*n* = 21). The data are represented as scatter plots with each circle representing a single individual. P values were calculated using the Mann-Whitney test with Holm's correction for multiple comparisons.

To assess if there is an association between the systemic levels of pro-inflammatory cytokines, chemokines, growth factors and disease severity in PIMS-TS, we compared the circulating levels of inflammatory markers between PIMS-TS children needing PICU care (*n* = 23) and children who did not require PICU care (*n* = 21) and observed that the systemic levels of IFNγ, TNFα,  IL-6, IFNβ  and IL-10 were significantly higher in children with PIMS-TS needing PICU care (Fig. 5), suggesting a correlation between elevated systemic levels of pro-inflammatory cytokines and clinical severity.

### 3.7 Plasma cytokines are markers of disease severity in COVID-19

**Fig. 6:**
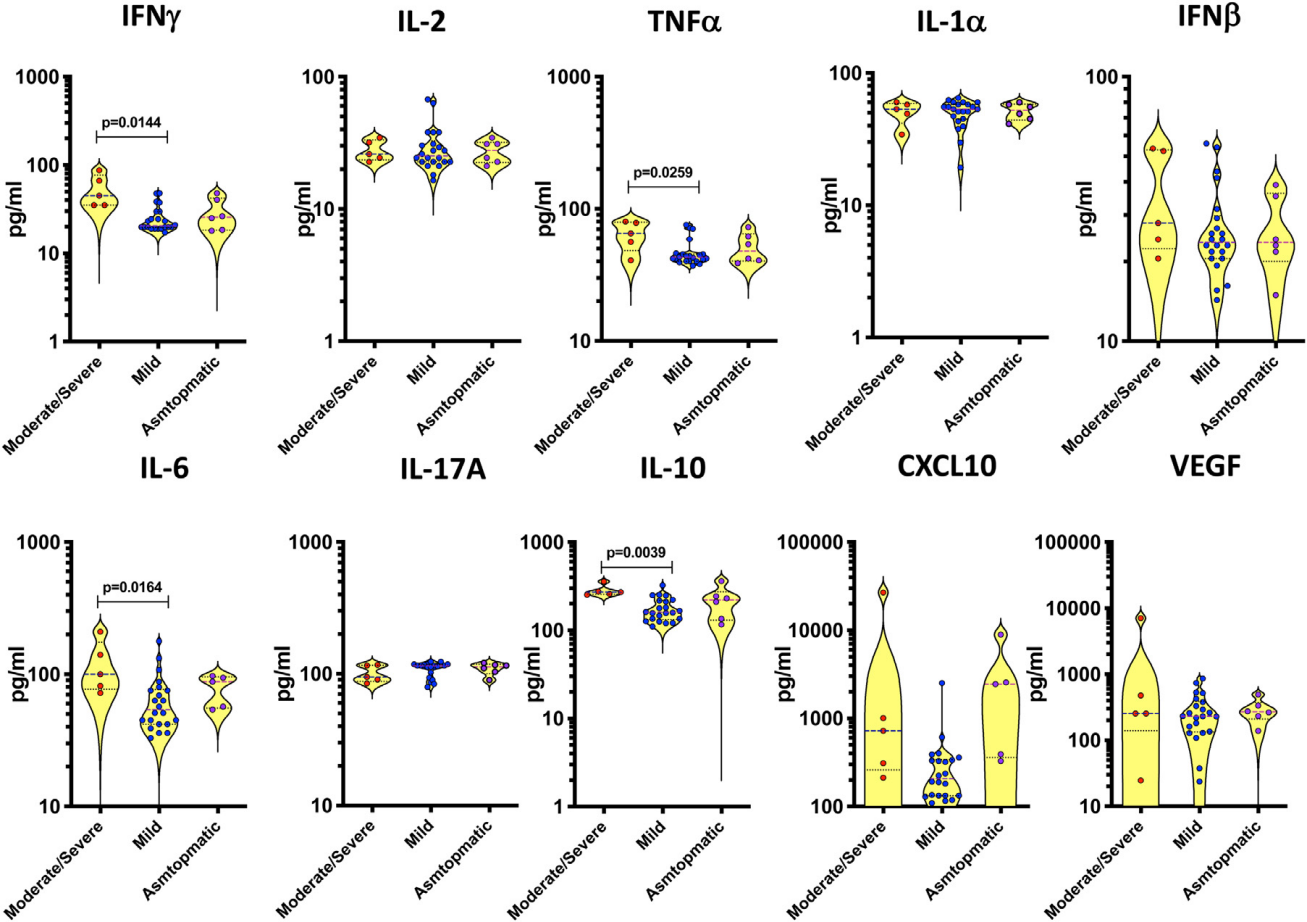
Circulating levels of cytokines, chemokines and growth factors in children with asymptomatic, mild and moderate to severe COVID-19 The plasma levels of IFNγ, IL-2, TNFα, IL-1α, IFNβ, IL-6, IL-17A, IL-10, CXCL10 and VEGF were measured in children with moderate to severe (*n* = 5), children with mild (*n* = 22) and asymptomatic (*n* = 5). The data are represented as scatter plots with each circle representing a single individual. P values were calculated using the Kruskal—Wallis test with Dunn's post—hoc for multiple comparisons.

Next, we evaluated the correlation between the circulating levels of inflammatory markers and disease severity in COVID-19 and found that the systemic levels of TNFα,  IL-6, IL-10 and CXCL10 were significantly higher in children with moderate to severe disease in comparison to children with mild disease (Fig. 6).

### 3.8 Correlation of plasma immune markers with C - reactive protein (CRP), lymphocyte count, sodium, D-Dimer, ferritin and LDH

**Fig. 7:**
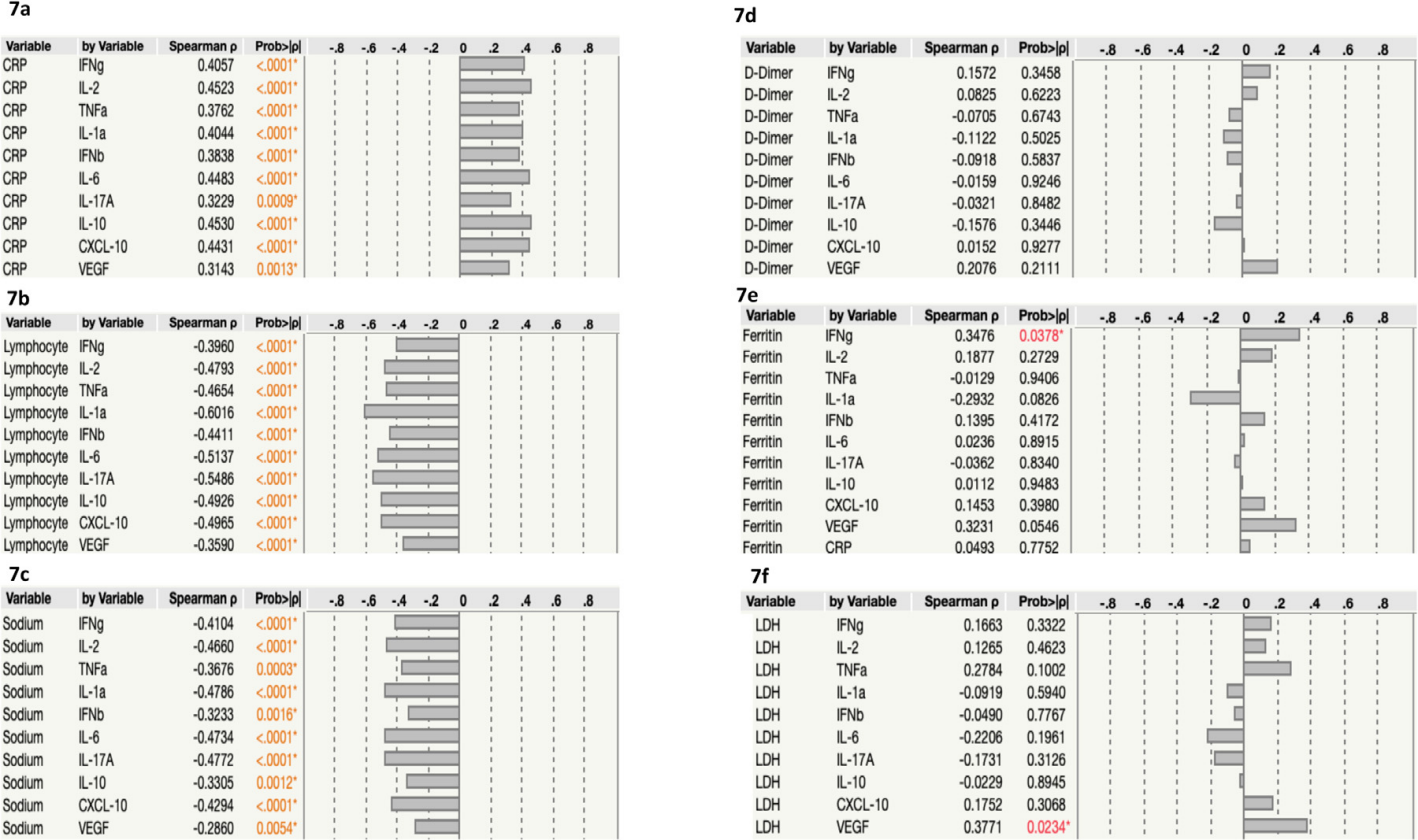
Relationship between immune markers and C - reactive protein (CRP), Lymphocyte count, sodium, D-Dimer, ferritin and LDH. The relationship between the plasma levels of immune markers vs (a) CRP (b) lymphocyte count (c) sodium (d) D-Dimer (e) Ferritin and (f) LDH in all PIMS-TS, COVID-19 and seropositive. P and r values were calculated using the Spearman Rank Correlation.

We performed Spearman rank correlation analysis to determine the association of plasma immune markers with CRP, Lymphocyte count, sodium, D-Dimer, ferritin and LDH. CRP was positively associated with IFNγ,  IL-2, TNFα IL-1α IFNβ IL-6 IL-17A IL-10, CXCL-10 and VEGF in children with PIMS-TS and COVID-19 (Fig. 7A). Lymphocyte count was negatively associated with IFNγ, IL-2, TNFα,  IL-1α, IFNβ,  IL-6, IL-17A, IL-10, CXCL-10 and VEGF in children with PIMS-TS and COVID-19 (Fig. 7B). Likewise, we found that sodium levels were negatively associated with IFNγ, IL-2, TNFα, IL-1α, IFNβ, IL-6, IL-17A IL-10, CXCL-10 and VEGF (Fig. 7C). We did not find any significant correlation between D-Dimer, ferritin and LDH levels with immune markers.

## 4 Discussion

We describe the immune profile of children presenting with different spectrum of SARS-CoV-2 infection, ranging from acute COVID-19 disease to PIMS-TS, and identify immune biomarkers that could help differentiate the varied presentation of infection. Elevated levels of cytokines may have systemic effects and may in turn cause organ dysfunction [12]. This may potentially be one of the reasons for children with PIMS-TS presenting with multi-organ dysfunction. Results from our study provide novel data that might further improve our understanding of the immunology and pathogenesis of PIMS-TS as well as COVID-19. We observed that the immune profile of children with PIMS-TS was distinct form that of COVID-19 and seropositive children, with markedly elevated cytokines and chemokines. This is similar to the data from other groups analysing the immune responses in children with PIMS-TS, [4–7] however unlike previous studies, we report an extended cytokine profile.

Initial reports emphasised and considered PIMS-TS as an atypical form of Kawasaki Disease (KD) [2,13]. The symptomology of PIMS-TS and KD is indeed overlapping and similar, [2,3,13] presenting as a diagnostic challenge and it is extremely difficult to differentiate the cytokine storm of PIMS-TS and KD on the basis of clinical features alone [6,12,14]. Immunological assessment, therefore, could help unravel disease severity and establish the clinical course in PIMS-TS. Both TNFα and IL17A are known to be associated in the pathogenesis of PIMS-TS and KD [4,15]. However, levels of IFN-γ which is not known to be strongly associated with KD is markedly increased in PIMS-TS as well as COVID-19, [4–6] signifying that IFNγ may be important in the pathogenesis of PIMS-TS and COVID-19, thus implying the role of T- cells, NK- cells and macrophages. Likewise, marked elevation in IL-10 is associated with PIMS-TS which is uncommon in KD, where we tend to observe high levels of IL-1, IL-2, and IL-6 [4,15]. In concordance, children with PIMS-TS in our study had markedly increased levels of IFNγ and IL-10. Correlating with the elevated levels of cytokines and chemokines, gastrointestinal symptoms and myocardial dysfunction were more prevalent in PIMS-TS children, both of which are not commonly seen in KD. Results of our study adds to the evidence that the cytokine profile of PIMS-TS is different from that of previously described in KD, [4], [5], [6], [7] thus emphasising that PIMS-TS is a distinct paediatric illness.

We also observed differences in the immune profile of children with PIMS-TS needing PICU care and those who did not require PICU care (Table 3). PIMS-TS children with shock requiring vasoactive medications were admitted to PICU and were characterised by elevated levels of INFγ, TNFα, IL-6 and IL-10, affirming the distinct immunopathogenesis of PIMS-TS. Similarly, we also found differences in the immune profile of children with moderate to severe COVID-19 and mild COVID-19, suggesting a correlation between elevated systemic levels of pro-inflammatory immune markers and disease severity.

**Table 3:** Comparison of children with PIMS-TS needing PICU care and no PICU care.

	PIMS-TS admitted to PICU *n* = 23	PIMS-TSNo PICU *n* = 21	p value
**Age**	7.2y (11 m – 16 y)	5y (6 m – 13 y)	0.116
**Male**	11 (37%)	14 (56%)	0.16
**Clinical features n (%)**			
Fever	23 (100%)	21 (100%)	
Gastrointestinal symptoms	18 (78%)	19 (90%)	0.2
Mucocutaneous symptoms	17 (74%)	17 (81%)	0.5
Respiratory symptoms	9 (39%)	2 (9%)	0.006
Hypotension	23 (100%)	0	<0.00001
Shock (vasodilated)	18 (78%)	3* (14%)	<0.00001
**ECHO at PICU admission (n)**			
*Coronary artery dilatation*	2	0	
*Myocardial dysfunction*	23	0	<0.00001
**Respiratory support (n)**			
Nasal cannula oxygen	1	6	
HHFNC	2	0	
Mechanical ventilation	0	0	
**Cardiovascular support (n)**			
Fluid bolus	21 (91%)	3 (14%)	
Vasoactive support	19 (83%)	0	
*Adrenaline*	7		
*Nor-adrenaline*	19		
**Medications n (%)**			
IVIG	23 (100%)	14 (66%)	
Steroids	23 (100%)	9 (43%)	
Immunomodulatory agent#	1	1	
Aspirin	17 (75%)	0	
Antibiotics	23 (100%)	21 (100%)	
Gastro protective agents	23 (100%)	21 (100%)	
**Laboratory parameters**			
***CRP (<5*** ***mg/L)***	177 (37.5 - 298)	137 (9 – 370)	0.34
***D-Dimer (ng/ml FEU)*** ***(100–500) median (IQR)***	4890 (2446 – 10,000)	3718 (117 – 10,000)	0.03
***Lymphocyte(/mm3)*** ***(1500 – 4000) median (IQR)***	1386 (330 – 4540)	2023 (880 – 6460)	0.13
***Neutrophils (/mm3)*** ***(1500 – 7000) median (IQR)***	7800 (3360 – 14,180)	9500 (2700 – 34,000)	0.08
***Sodium (135–***145 mmol***/l) median (IQR)***	133 (130 – 136)	133 (127 −143)	0.44
***Ferritin (ng/mL)*** ***(7 to 140) median (IQR)***	605 (38 −2571)	247 (101 – 1104)	0.006
***LDH (U/L)*** ***(125–243) median (IQR)***	494 (200 −905)	487 (264 −928)	0.3

⁎ 3 children required fluid bolus at ER.

# Immunomodulatory drug – Tocilizumab.

Children with PIMS-TS in our cohort were treated with intravenous immunoglobulins (IVIG), corticosteroids, and IL-6 inhibitor (Tocilizumab). IVIGs can neutralize the immunological effect of autoantibodies and use of steroids may provide broad immunosuppression, however we are yet to fully understand the immumopathological role of these treatments in PIMS-TS [16,17]. Currently there is no consensus on choice of biological agents (IL-1 antagonists vs IL-6 receptor blockers vs anti-TNF agents) in the management of PIMS-TS, [17] which is based on the clinicians discretion. Interestingly, our cohort of children did not have high levels of IL-1β, implying that further research is warranted to establish whether IL-1 antagonists can be used as a potential therapy in PIMS-TS among Indian children. However, IL-1 antagonists, IL1RA (anakinra), is currently unavailable in India and IL-6 receptor blocker, Tocilizumab has been used in a few children [10,18]. We also observed that children with PIMS-TS in our cohort had few underlying comorbidities as compared to COVID-19 and seropositive children (Tables 1 and 2). The extent to which this observation influences the pathogenesis is not known and requires further investigations with a focus on genetic studies.

It has been well known that cytokines play an important role in immunopathology during viral infection [12]. Recent published studies in adult populations have reported that elevated levels of inflammatory cytokines levels such as TNF-α, IL-1β, IL-6, IL-10, IL-17, IL-18, IFN-γ are seen in active COVID-19 individuals compared to seropositive individuals and healthy controls [19,20]. TNFα and IFNγ are known to particularly drive COVID-19 disease severity [20] and in addition, IL-6, IL-1β and IL-12 have been consistently implicated in severe disease [19,20]. Similar to this finding from the adult population, children with COVID-19 in our study also showed distinct cytokine/chemokine upregulation in comparison to seropositive and control children. Hyperinflammatory response in PIMS-TS, albeit with a delayed presentation appears to be similar to that seen in severe COVID-19 in adults [6,19–21]. However, there are various immunological differences [22,23]. The cytokine storm in adult COVID-19 is characterised by increased IL-1, IL-6 and TNF-α, [19,20,24] and predominantly involves the respiratory system, whereas the cytokine storm in PIMS-TS, with elevated IFNγ, TNFα, IL-6, IL-10 and IL-17A, [4], [5], [6] involves multiple systems mainly gastrointestinal, mucocutaneous and cardiac, with few or no respiratory manifestations [3,22]. Congruent with this, the immune profile observed in PIMS-TS children showed prominent mucosal and endothelial immune signatures.

We also observed that the immune profile of non PIMS-TS seropositive children were notably different from the uninfected control children, indicating an association between the cytokines and the time from infection. The median duration of blood sampling in asymptomatic seropositive children was 3 weeks since proven or suspected COVID-19 illness or contact. Our study has limitations as we included children from a single institution with small number of children in all groups and we were unable to assess the immunological profile with longitudinal follow up. In addition, it would have been ideal to compare the immunological profile with that of children with KD from similar population during the same time. We also studied a smaller number of children with COVID-19, and are unable to describe and identify subtle differences in the immune profile of children with mild, moderate and severe disease. Future work investigating the role of genetics and other environmental factors in the development of severe COVID-19 and PIMS-TS is pivotal as this has the potential to guide treatment.

In conclusion, our study is the first study describing the immune profile of children with PIMS-TS and COVID-19 from a LMIC. We conclude, PIMS-TS is a distinct and unique immunopathogenic paediatric illness related to SARS-CoV-2, presenting with cytokine storm different from acute COVID-19 and other hyperinflammatory conditions. We were also able to provide biosignatures that differentiates asymptomatic seropositive children from uninfected, control children.The detailed analysis in our study provides additional information on the pathogenesis and may guide clinicians in the assessment and management of children with PIMS-TS and COVID-19 in future.

## Declaration of Competing Interest

The authors disclose no conflicts of interest

## Appendix Supplementary materials

Image, application 1
